# N-Terminal Truncated Intracellular Matrix Metalloproteinase-2 Induces Cardiomyocyte Hypertrophy, Inflammation and Systolic Heart Failure

**DOI:** 10.1371/journal.pone.0068154

**Published:** 2013-07-16

**Authors:** David H. Lovett, Rajeev Mahimkar, Robert L. Raffai, Leslie Cape, Bo-Qing Zhu, Zhu-Qiu Jin, Anthony J. Baker, Joel S. Karliner

**Affiliations:** 1 Department of Medicine, San Francisco Department of Veterans Affairs Medical Center, University of California San Francisco, San Francisco, California, United States of America; 2 Department of Surgery, San Francisco Department of Veterans Affairs Medical Center, University of California San Francisco, San Francisco, California, United States of America; 3 Cardiovascular Research Institute, University of California San Francisco, San Francisco, California, United States of America; UAE University, Faculty of Medicine & Health Sciences, United Arab Emirates

## Abstract

Matrix metalloproteinase-2 (MMP-2) is increasingly recognized as a major contributor to progressive cardiac injury within the setting of ischemia-reperfusion injury and ischemic ventricular remodeling. A common feature of these conditions is an increase in oxidative stress, a process that engages multiple pro-inflammatory and innate immunity cascades. We recently reported on the identification and characterization of an intracellular isoform of MMP-2 generated by oxidative stress-mediated activation of an alternative promoter located within the first intron of the MMP-2 gene. Transcription from this site generates an N-terminal truncated 65 kDa isoform of MMP-2 (NTT-MMP-2) that lacks the secretory sequence and the inhibitory prodomain region. The NTT-MMP-2 isoform is intracellular, enzymatically active and localizes in part to mitochondria. Expression of the NTT-MMP-2 isoform triggers Nuclear Factor of Activated T-cell (NFAT) and NF-κB signaling with the expression of a highly defined innate immunity transcriptome, including Interleukin-6, MCP-1, IRF-7 and pro-apoptotic transcripts. To determine the functional significance of the NTT-MMP-2 isoform in vivo we generated cardiac-specific NTT-MMP-2 transgenic mice. These mice developed progressive cardiomyocyte and ventricular hypertrophy associated with systolic heart failure. Further, there was evidence for cardiomyocyte apoptosis and myocardial infiltration with mononuclear cells. The NTT-MMP-2 transgenic hearts also demonstrated more severe injury following ex vivo ischemia-reperfusion injury. We conclude that a novel intracellular MMP-2 isoform induced by oxidant stress directly contributes, in the absence of superimposed injury, to cardiomyocyte hypertrophy. inflammation, systolic heart failure and enhanced susceptibility to ischemia-reperfusion injury.

## Introduction

We recently reported on the detailed characterization of an N-terminal truncated intracellular isoform of MMP-2 (NTT-MMP-2) generated by oxidative stress-mediated activation of an alternative promoter in the first intron of the MMP-2 gene [1]. Transcription from the alternative intronic promoter generates a truncated mRNA transcript wherein translation is initiated at a highly conserved Kozak consensus sequence (Methionine^77^) located within the second exon of the MMP-2 gene. The N-terminal truncated translation product encodes a MMP-2 isoform of 65 kDa molecular mass which lacks the secretory sequence and the inhibitory prodomain [2]. The NTT-MMP-2 isoform has an exclusively intracellular localization within both cystosolic and mitochondrial compartments and is intrinsically enzymatically active.

The 65 kDa MMP-2 isoform was initially detected in mitochondria-enriched preparations from aging wild type mice and in mitochondrial preparations of hypomorphic ApoE mice expressing an ApoE-like form of mouse ApoE (also referred to as “HypoE” mice). These mice are also deficient in the SF-B1 receptor (ApoER61^h/h^/SR-B1^−/−^ mice) and represent a model of diet-induced coronary atherosclerosis and myocardial infarction [1], [3]. Furthermore, the NTT-MMP-2 isoform was detected within mitochondrial preparations from cardiac-specific transgenic mice expressing the full length, secreted 68 kDa constitutively active isoform of MMP-2 in the setting of advanced ventricular systolic failure [1], [4].

In vitro studies with model embryonic cardiomyoblast H9C2 cells indicated that the NTT-MMP-2 isoform is induced by hypoxia or following chemical simulation of transient ischemia-reperfusion injury [1]. We further demonstrated that the NTT- MMP-2 isoform initiated mitochondrial-nuclear stress signaling via activation of Nuclear Factor of Activated T-cells (NFAT), NF-κB and Interferon Response Factor-7 (IRF-7) transcriptional networks. Microarray analysis of H9C2 cells transfected with the NTT-MMP-2 isoform identified a highly selective transcriptome composed almost exclusively of the innate immunity, chemokine and pro-apoptotic ontologies [1].

These initial studies, using both mice and model H9C2 cardiomyoblasts, generated the hypothesis that hypoxia or oxidative stress stimulates the synthesis of an NTT-MMP-2 isoform that directly contributes to progressive cardiac injury. We hypothesized that cardiac-specific expression of the NTT-MMP-2 isoform, in the absence of superimposed injury, would lead to the development of cardiomyocyte and ventricular hypertrophy through activation of NFAT and NF-κB signaling. Further, we hypothesized that expression of the NTT-MMP-2 isoform, through activation of NF-κB and IRF-7 signaling, would activate a primary innate immune response, with the development of inflammation, cardiomyocyte apoptosis and ventricular systolic failure. As detailed in this report, the cardiac-specific transgenic model manifested each of the hypothesized features, thereby confirming a central and independent role for the NTT-MMP-2 isoform in ischemic cardiac disease.

## Results

### Generation and characterization of cardiac-specific N-terminal truncated MMP-2 transgenic mice

**Figure 1:**
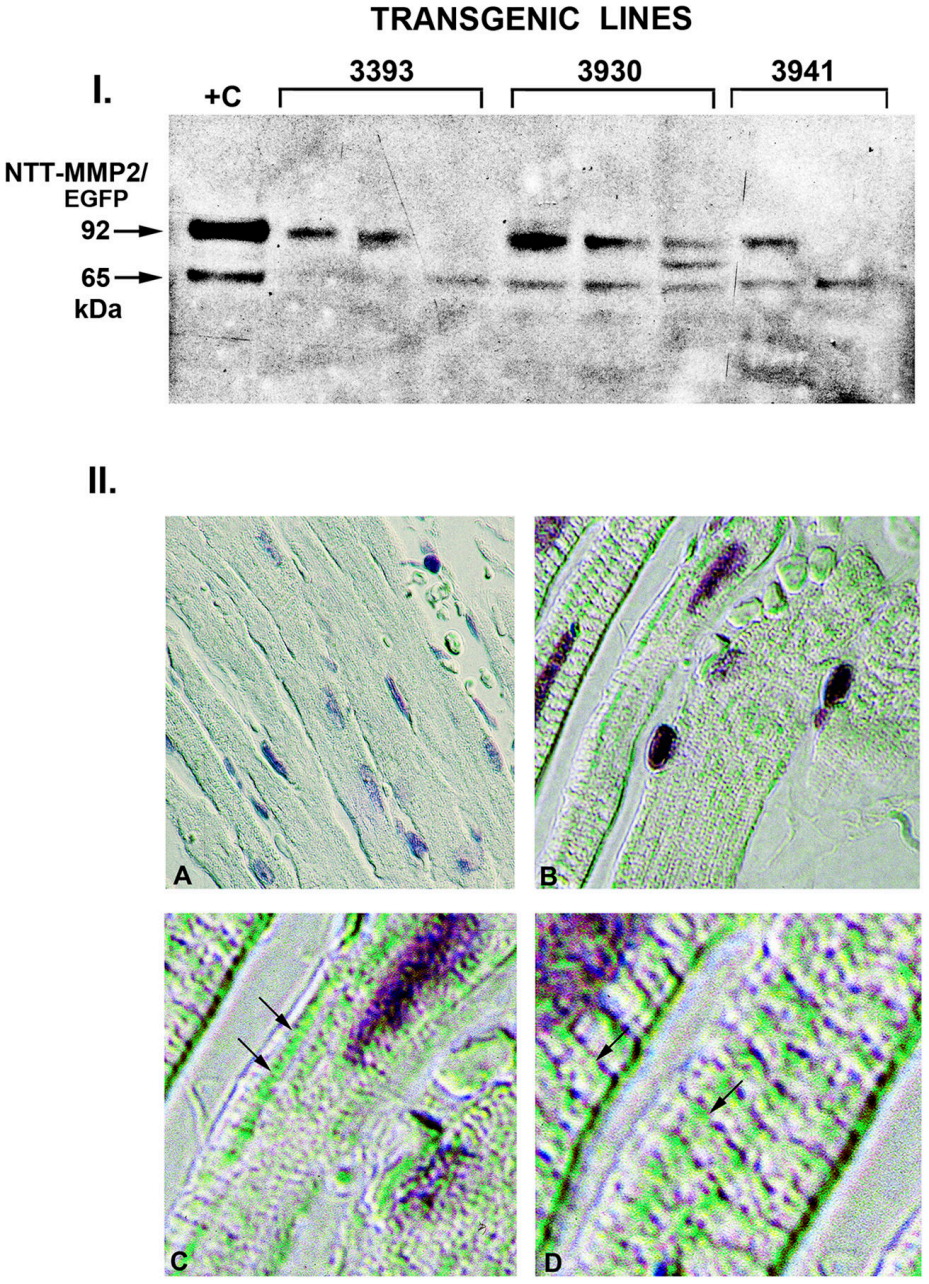
Expression of the NTT-MMP-2/EGFP fusion protein in transgenic hearts. I. Western blot (using anti-MMP-2 IgG) of ventricular mitochondrial lysates isolated from 2–3 progeny of three individual transgenic founder lines (3393, 3930 and 3941). The NTT-MMP-2/EGFP fusion protein has an apparent molecular mass of 92 kDa and there is evidence for proteolytic cleavage of the EGFP component in several of the hearts, leaving the 65 kDa NTT-MMP-2 protein intact (+C: recombinant NTT-MMP-2/EGFP fusion protein). II. Immunohistochemistry of wild type (WT, panel A) and transgenic (TG, panels B–C) ventricular sections probed with anti-EGFP antibody and examined using Nomarksi optics. Compared to the WT controls, immunostaining is present in dense clusters (green pseudocolor) extending longitudinally between the myofilaments. In addition, linear arrays of reaction product are present in a subsarcolemmal distribution (panel C, arrows) as well as perpendicularly across the long axis of individual cardiomyocytes (panel D, arrows). The staining distribution is consistent primarily with a mitochondrial localization of the NTT-MMP/EGFP protein. (Final magnification: A, B: X325; C: X600; D: X900).

In order to determine the pathophysiologic significance of the N-terminal truncated MMP-2 protein within the context of the intact heart, we generated cardiac-specific transgenic mice as detailed in Materials and Methods. The expression cassette consisted of the N-terminal truncated MMP-2 cDNA with the addition of a C-terminal EGFP tag to distinguish the transgenic product from intrinsic MMP-2 protein. We note that EGFP has been previously successfully used within cardiac and other transgenic contexts and, in the absence of extreme over-expression, does not induce cellular toxicity [5]–[9]. We identified nine founders and performed initial characterization of mice derived from three independent lines chosen for low (<3) numbers of transgene copies. Representative Western blot analyses (Figure 1, panel I.) of ventricular mitochondrial extracts probed for MMP-2 revealed the expected 92 kDa N-terminal truncated MMP-2/EGFP fusion protein. There was evidence for proteolytic cleavage of the EGFP tag in some extracts. This also occurred with purified recombinant NTT-MMP-2/EGFP protein in vitro, which is due to active MMP-2-mediated autocatalytic cleavage [10], [11]. Given the inherent autocatalytic activity of MMP-2 lacking the prodomain, it is likely that the cellular half-life of the expressed transgenic protein is relatively short.

To directly quantify the amount of expressed transgenic protein we performed scanning densitometric analyses of Western blots of ventricular extracts from the three evaluated founder lines and compared these with signal intensities obtained with known quantities of purified recombinant N-terminal truncated MMP-2/EGFP protein. Under these conditions the transgenic mice expressed a mean of 3.14±0.96 ng EGFP protein/100 µg ventricular lysate protein (n = 6 hearts). This corresponds to an EGFP protein concentration of 0.11 pM, an amount considerably lower than that measured in other EGFP transgenic models without evident cellular toxicity [8], [9]. This amount of expressed transgene is lower than the amount of endogenously generated NTT-MMP-2 protein we detected in ventricular lysates from ageing and hypomorphic ApoER61^h/h^ /SR-BI KO mice, indicating that the transgenic cardiac phenotype reported here is not the consequence of gross transgene overexpression. The cardiac-specific NTT-MMP-2 mice are born in a Mendelian distribution, are fertile, normotensive and live approximately 13–14 months when they die of systolic heart failure. Four month old transgenic mice are robust and have normal basal cardiac physiology as detailed below.

### Histologic analyses of N-terminal truncated MMP-2 transgenic hearts-mitochondrial transgene localization and development of cardiomyocyte hypertrophy

We performed immunohistochemistry of control and transgenic ventricular sections (lines 3393 and 3930 in which the NTT-MMP-2/EGFP fusion protein was relatively intact, Figure 1) using a monoclonal antibody to the EGFP tag to determine the intracellular localization of the transgenic protein. In our prior studies of NTT-MMP-2 protein localization in the cardiac embryonic cardiomyoblast H9C2 cell line, we determined that approximately one-third of the NTT-MMP-2 protein was present within the mitochondrial intramembranous space and approximately two-thirds was present within a highly purified cytosolic fraction [1]. Notably, H9C2 cells lack an organized sarcomeric contractile apparatus but do contain abundant mitochondria. As detailed in Figure 1, panel II., the immunohistochemical reaction product (displayed in green pseudocolor to enhance contrast, original image provided as Figure S1) was not randomly distributed throughout the cardiomyocytes but was concentrated in a punctate pattern arranged in linear arrays throughout the cells (Panel B). Examination at higher magnification localized the punctuate reaction product primarily to longitudinal arrays between myofilaments, which were not decorated with reaction product. These results are consistent with a mitochondrial rather than sarcomeric localization (panel C). In addition, some cardiomyocyte sections (panel D) displayed apparent horizontal arrays of reaction product extending across myofilaments, possibly consistent with a sarcoplasmic localization (D).

**Figure 2:**
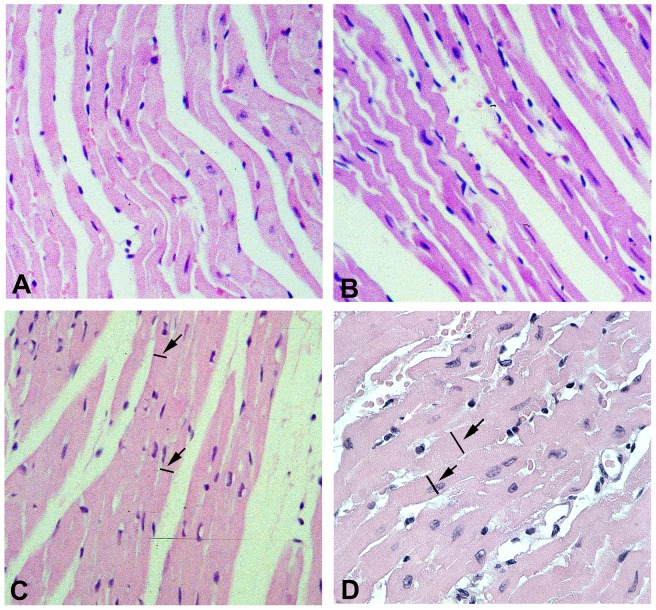
Conventional histologic analysis of WT and NTT-MMP-2 transgenic hearts at four months and six months of age. Hematoxylin/eosin-stained sections of left ventricular free walls from four month old WT (panel A) and NTT-MMP-2 transgenic mice (panel B) demonstrate normal cardiomyocyte structure and organization. By six months of age, the transgenic hearts (cf. panel C, WT and panel D, TG) have developed cardiomyocyte hypertrophy (arrows point out cross-sectional markers) and a loss of cardiomyocyte organization (X300).

Conventional hematoxylin/eosin-stained ventricular sections of the transgenics at four months of age revealed normal structure as compared to wild type litter mate controls (Figure 2, cf. panels A and B). By 6 months of age ventricular sections from the N-terminal truncated MMP-2 transgenics revealed cardiomyocyte hypertrophy as manifested by increased cross-sectional areas. (Figure 2, cf. panels C and D). Cardiomyocyte cross-sectional areas were 176±16 µm^2^ in the wild type mice and 241±14 µm^2^ in the NTT-MMP-2 transgenic mice (P<0.05, n = 6 ventricles/study group). In addition to cardiomyocyte hypertrophy, the architectural organization of the cardiomyocytes was disordered.

### Ultrastructural analysis defines disordered cardiomyocyte contractile apparatus architecture and mitochondrial structure

**Figure 3:**
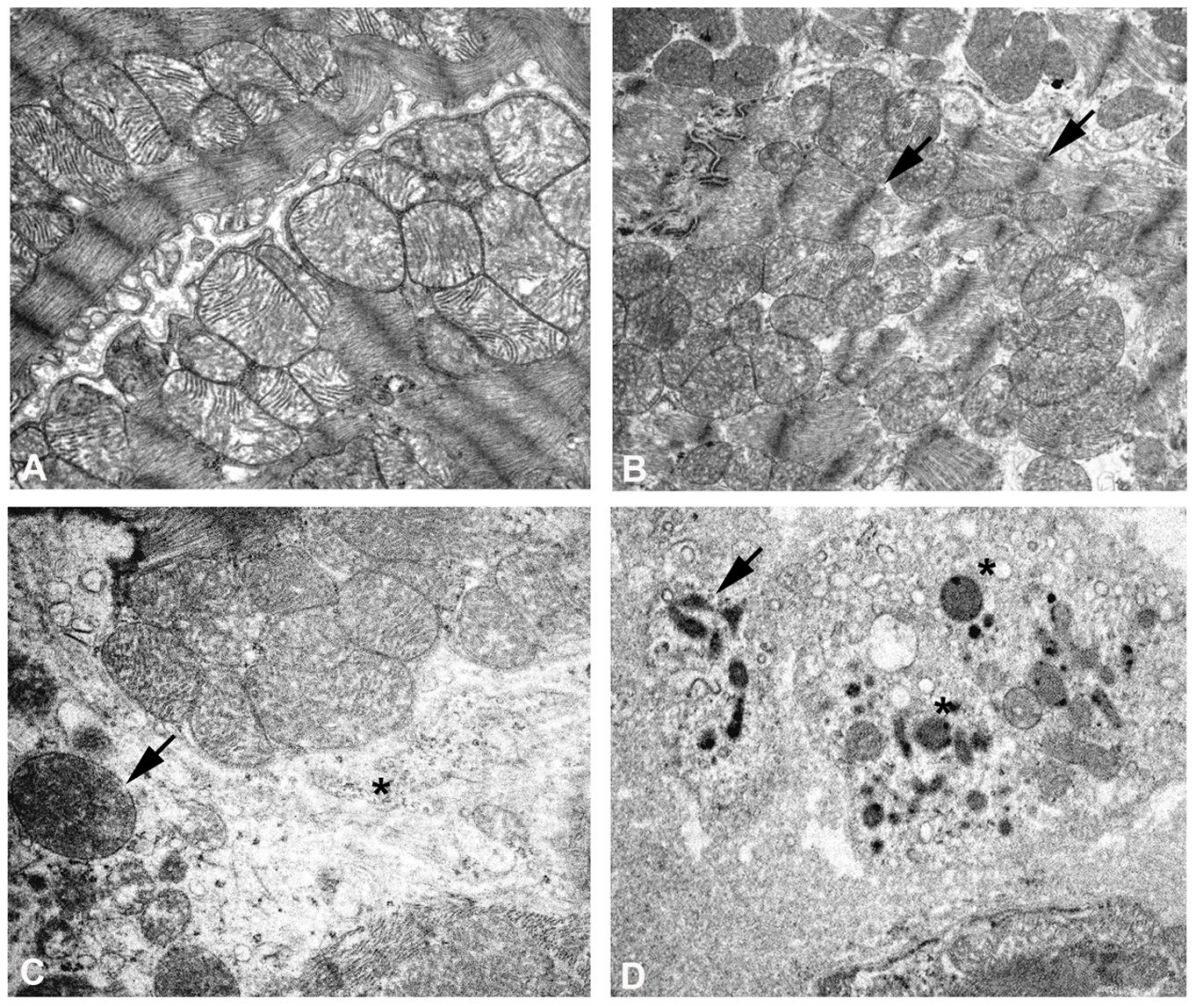
Ultrastructural analysis of WT and NTT-MMP-2 transgenic hearts at six months of age. Transmission electron microscopy of left ventricular free walls from WT (panel A) and NTT-MMP-2 transgenic hearts at 6 months of age (panels B-D). As compared to the WT controls, there is a clear loss of sarcomeric organization and Z-band registration in the NTT-MMP-2 transgenic cardiomyocytes. The mitochondria are heterogeneous in size and frequently swollen (panel C), with areas of organelle dropout (*). Further, there is ultrastructural evidence for autophagy (panel D), with abundant lysosomes (*) and autophagosomes (arrow). (X8000).

Transmission electron microscopy performed on six month old NTT-MMP-2 transgenics revealed considerable structural abnormalities as compared to the wild type controls (Figure 3). Myofilament architecture was disordered and there was loss of Z-band registration, but there was only limited overt myofilament lysis (cf. WT panel A, TG panel B). The mitochondria in the transgenic hearts were heterogeneous in size and frequently displayed loss of organized cristae (panel C). In addition, some transgenic cardiomyocytes underwent autophagy, as evidenced by the presence of characteristic lysosomes and autolysosomal structures (panel D).

### Older NTT-MMP-2 transgenic mice develop pronounced cardiomyocyte hypertrophy associated with apoptosis in the absence of replacement fibrosis

**Figure 4:**
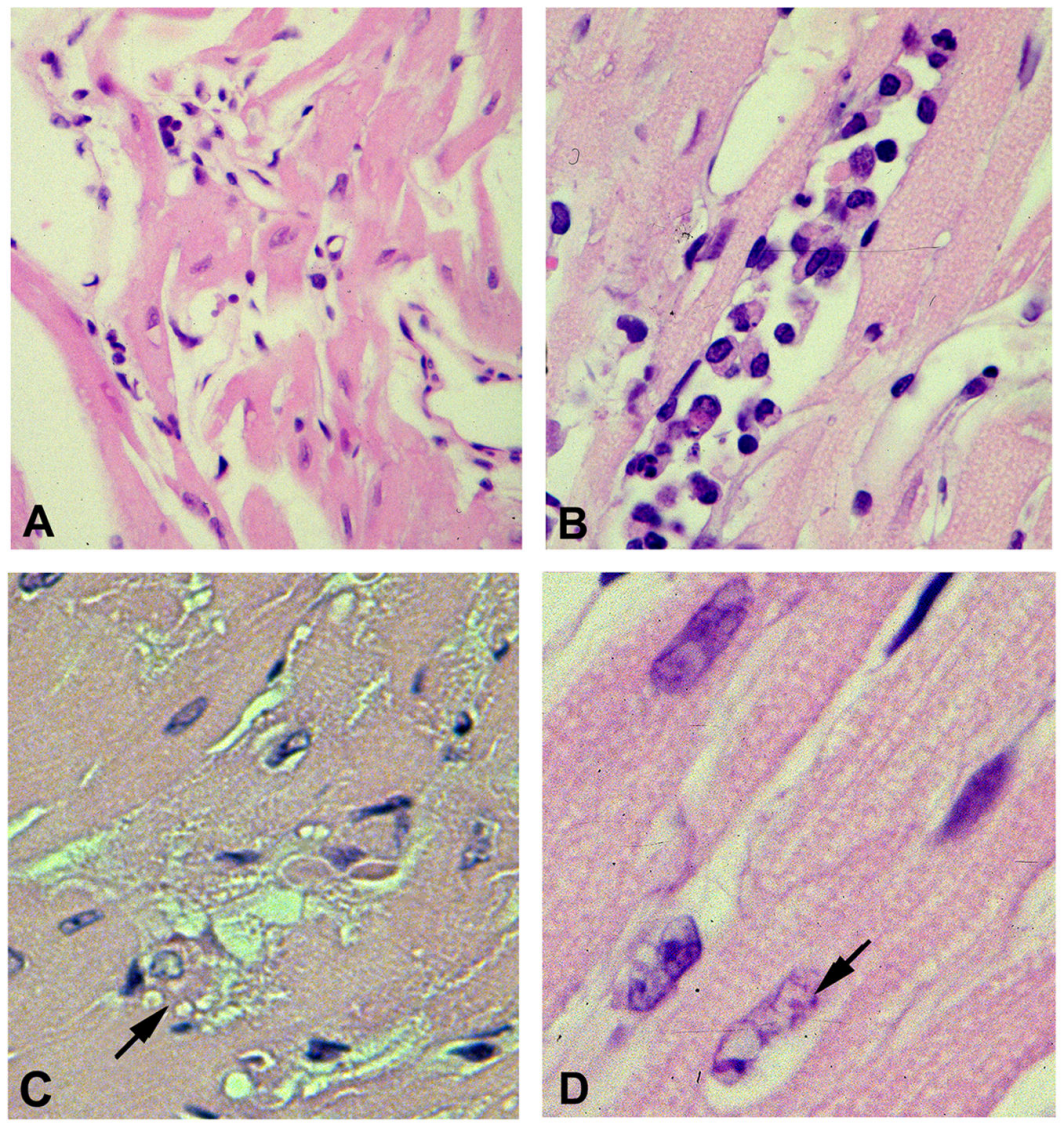
Cardiac NTT-MMP-2 expression induces progressive disruption of cardiomyocyte architecture, mononuclear cellular infiltration and apoptosis. Ventricular sections of 12 months old transgenic heart have grossly disordered cardiomyocyte architecture and diffuse mononuclear cell infiltration (panel A). There are frequent intense foci of mononuclear cell infiltration (panel B). Morphologic features of cardiomyocyte apoptosis, including cytoplasmic boiling (panel C, arrow) and nuclear chromatin peripheral condensation (panel D, arrow) are present. Final magnifications: A: X 300; B,C: X 600; D: X 900.

**Figure 5:**
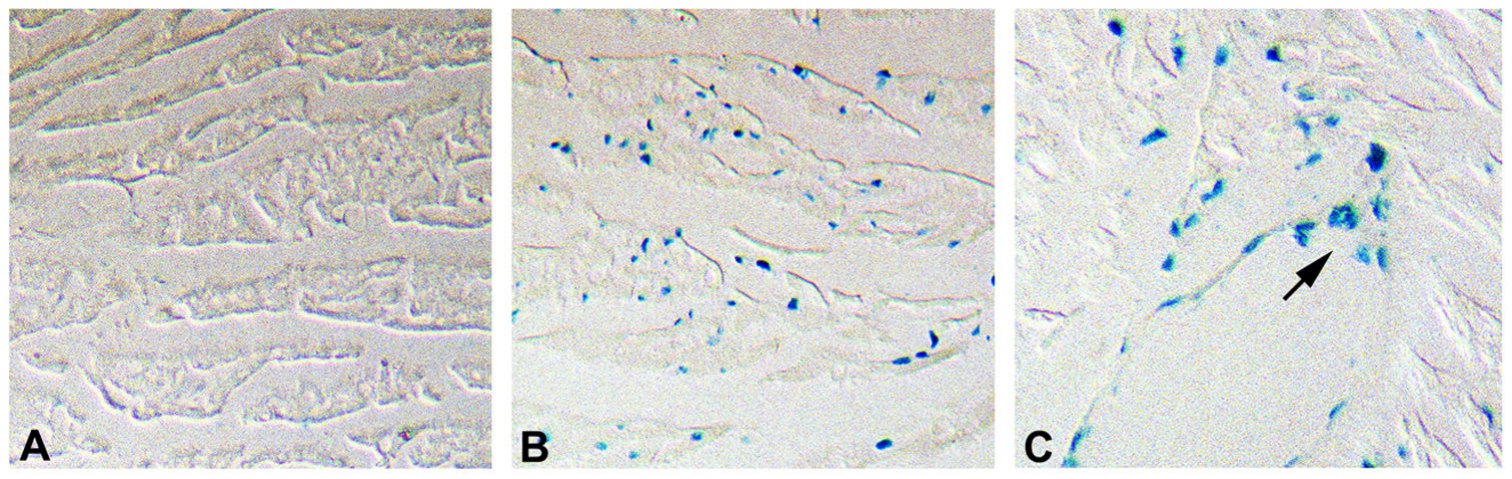
TUNEL staining confirms the morphologic evidence of cardiomyocyte apoptosis in NTT-MMP-2 transgenic hearts. TUNEL stain of twelve month old WT (panel A) and NTT-MMP-2 transgenic hearts (panels B, C) demonstrates diffuse cardiomyocyte apoptosis, (panel B), as well as endothelial cell apoptosis within a coronary artery (panel C, arrow). Final magnifications: A, B X 200; C X 300.

By 12 months of age the cardiomyocyte hypertrophy was much more pronounced and the cellular architecture was extensively distorted (Figure 4, panel A). There were numerous foci of mononuclear cell infiltration (panel B) that consisted primarily of T cells and monocytes on the basis of immunohistochemical staining for CD3 and CD11c (not shown). There was also morphologic evidence for cardiomyocyte apoptosis (Figure 4, panels C and D) as manifested by cytosolic vacuolization (cytoplasmic “boiling”) and perinuclear chromatin condensation [12], [13]. Cardiomyocyte apoptosis in 12 month old transgenic hearts was directly confirmed by TUNEL analysis of ventricular sections (Figure 5).

**Figure 6:**
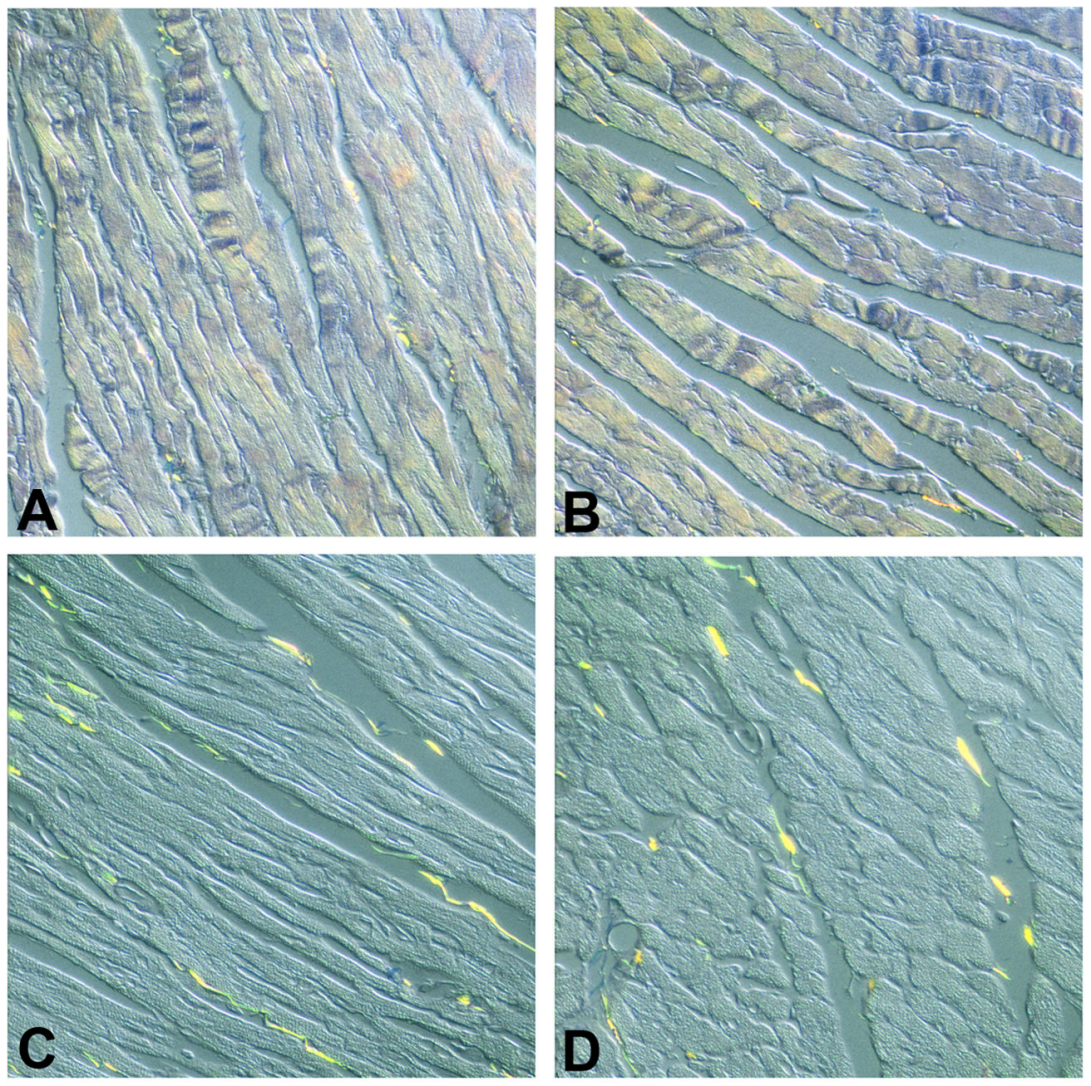
Assessment of cardiac interstitial collagen accumulation in WT and NTT-MMP-2 transgenic hearts. Picrosirius Red staining of four month old WT (panel A) and NTT-MMP transgenic ventricular sections (panel B). There is minimal interstitial collagen detected in either group. At twelve months of age there are minor, and equivalent, increases in interstitial collagen in the WT and NTT-MMP TG hearts cf. panels C, D). (X250).

We performed Picrosirius Red staining on sections of wild type and NTT-MMP-2 transgenic left ventricular free walls at 4 and 12 months to determine interstitial collagen content. Representative sections of wild type and N-terminal truncated MMP-2 transgenic hearts are detailed in Figure 6. The wild type and transgenic hearts had equivalent low levels of interstitial collagen at four months. At 12 months there was a small increase in interstitial collagen staining in the N-terminal truncated MMP-2 transgenic hearts, but this did not differ from that observed with the age-matched litter mate controls. Thus, in contrast to the extensive replacement fibrosis seen in transgenic mice expressing the full length secreted form of MMP-2, [4], expression of the NTT-MMP-2 isoform is not associated with increases in cardiac fibrosis. In further contrast with the full length MMP-2 transgenic mice we did not detect MMP-9,-13 or -14 in ventricular extracts from the NTT-MMP-2 transgenic mice (data not shown).

### Transgenic expression of the NTT-MMP-2 isoform is not associated with major myofilament lysis

**Figure 7:**
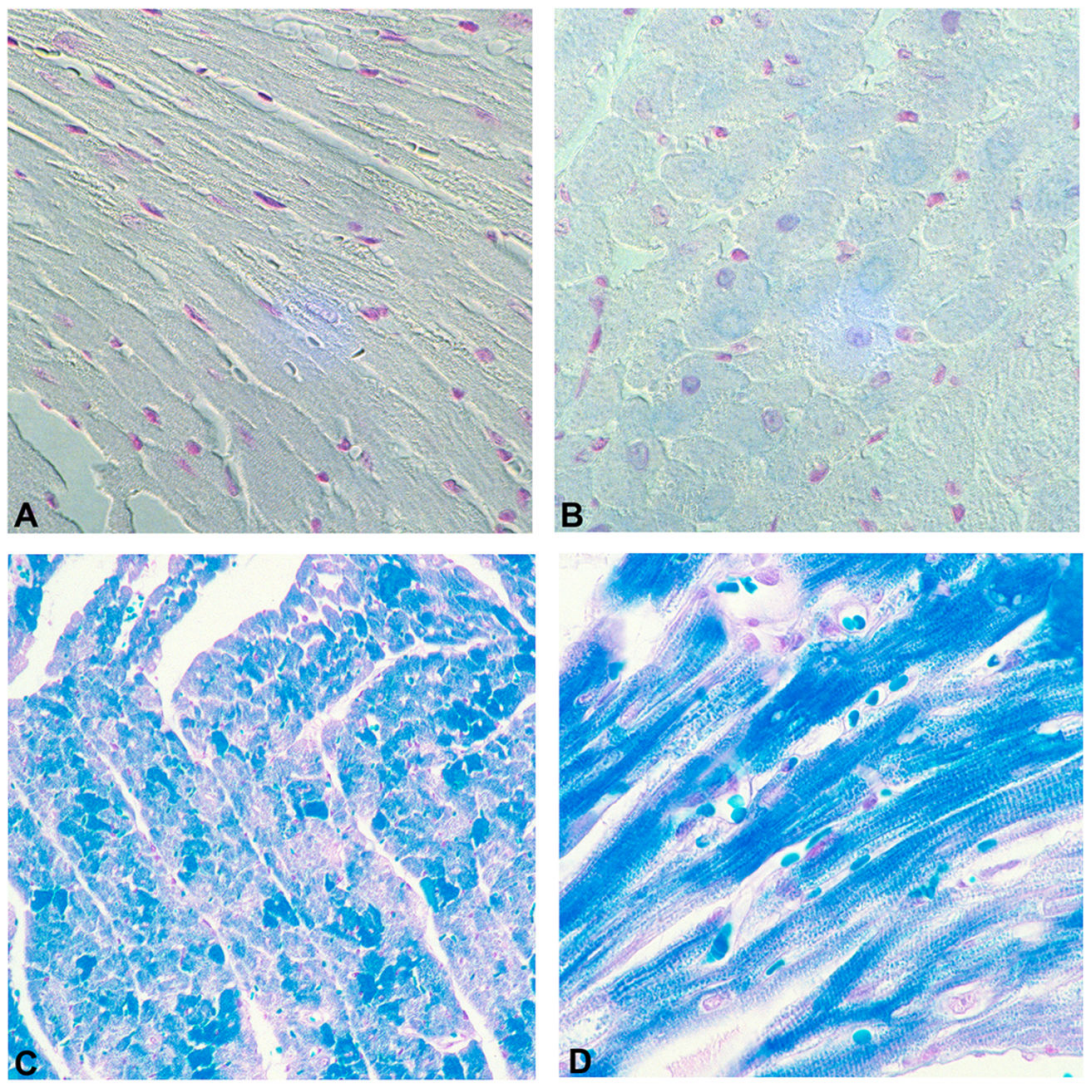
Luxol Fast Blue histochemical assessment of cardiomyocyte myofilament lysis. Luxol Fast Blue staining for the analysis of cardiomyocyte myofilament integrity was performed in left ventricular free walls from 12 month old WT (panel A), 12 month old NTT-MMP-2 transgenic mice (panel B) and 12 month old hearts expressing the full length MMP-2 cDNA (panels C and D). Myofilament lysis results in Luxol Fast Blue staining. There is little Luxol Fast Blue staining in the 12 month old WT or NTT-MMP transgenic hearts while there is abundant Luxol Fast Blue staining in the 12 month old full-length MMP-2 transgenic hearts, consistent with myofilament lysis. (X250).

We previously described myofilament lysis at the ultrastructural level and systolic failure in isolated hearts from mice expressing the full-length 68 kDa MMP-2 transgene [4], [14]. Isolated trabecular preparations from these mice demonstrated a primary contractile defect within the sarcomeric apparatus, consistent with the lysis of these structures observed by transmission electron microscopy [14]. In the present study we used Luxol Fast Blue staining of 12 month old full length MMP-2 transgenic mouse hearts and 12 month old NTT-MMP-2 transgenic mouse hearts to assess at the light microscopic level the extent of cardiomyocyte myofilament lysis [15], [16]. As shown in Figure 7, there was little evidence for cardiomyocyte myofilament lysis at the light microscopic level in ventricular sections of the N-terminal truncated MMP-2 transgenics (cf. panel A, WT, panel B, NTT-MMP-2 transgenic), while there was abundant Luxol Fast Blue staining of ventricular sections of full-length MMP-2 transgenics, (panels C, D), consistent with our reported myofilament lysis in this model. Preliminary studies with isolated trabeculae from the NTT-MMP-2 transgenic mice also do not demonstrate a primary sarcomeric contractile apparatus defect (data not shown). Thus, there is a major difference in myofilament integrity between the transgenic model expressing the full length enzymatically active MMP-2 protein and the model expressing the N-terminal truncated MMP-2 isoform.

### Noninvasive assessment of cardiac function in the NTT-MMP-2 transgenic mice reveals progressive ventricular hypertrophy and systolic heart failure

**Figure 8:**
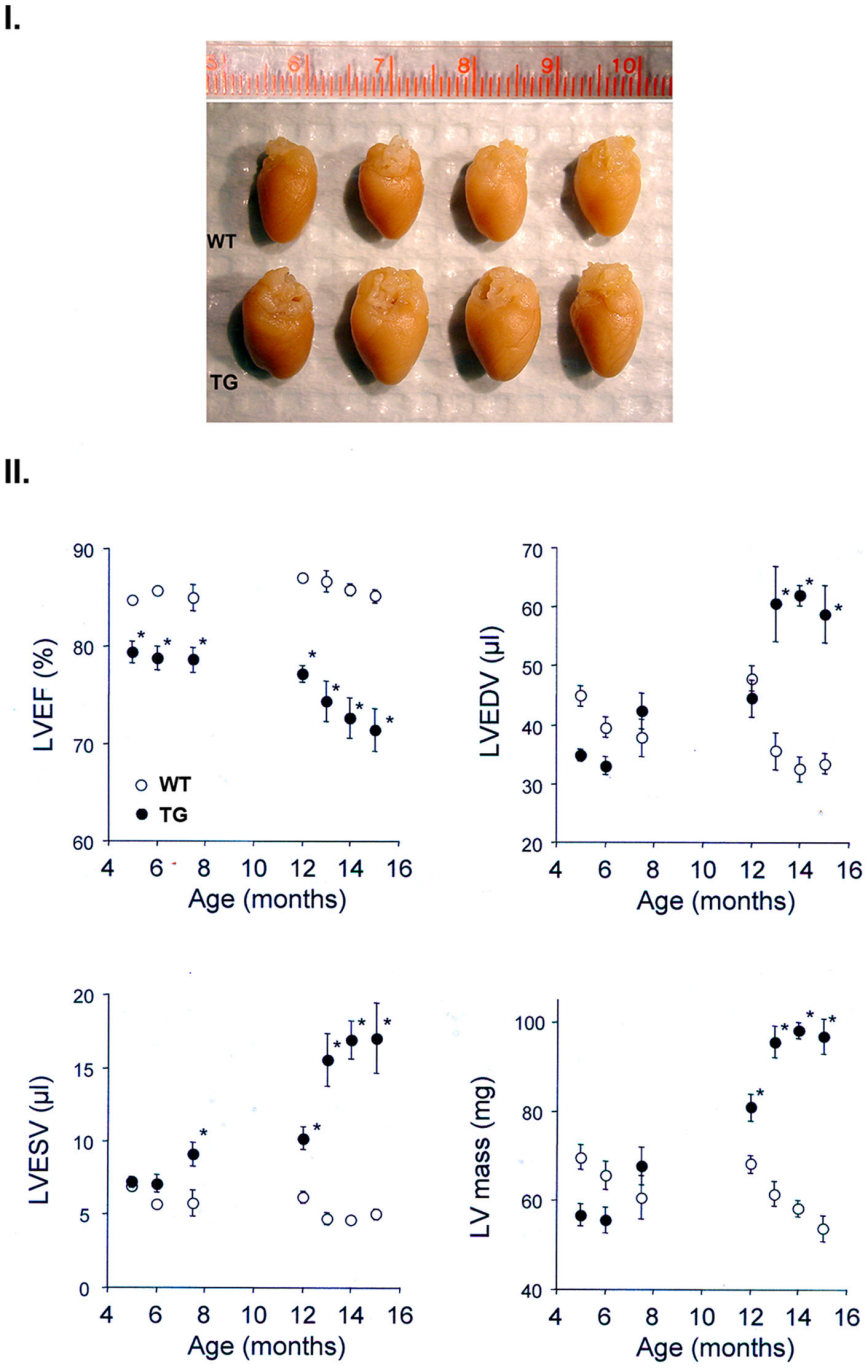
Gross cardiac morphology and serial non-invasive quantitation of cardiac function in NTT-MMP-2 transgenic mice. I. Formalin-fixed excised hearts from 12 month old WT and NTT-MMP transgenic mice. There is an approximate two-fold increase in the cardiac vertical and horizontal diameters in the NTT-MMP TG mice, which correlates with the measured increases cardiac mass. II. Serial changes in left ventricular ejection fraction (LVEF), LV end-diastolic volume (LVEDV), LV end-systolic volume (LVESV) and LV mass as determined by echocardiography are shown. Two cohorts of mice (WT and TG) were studied serially over 5-8 months of age and two other cohorts (WT and TG) were studied serially between 12 and 15 months of age. Comparisons were made at each time point using an unpaired t-test (n = 5–6 for each cohort; * P<0.05).

At the gross anatomic level, excised hearts from 12 month old NTT-MMP-2 transgenic mice were obviously hypertrophic (Figure 8, panel I.). Expressed as the ratios of cardiac weight (mg)/left tibial length (mm), the wild type mice had a ratio of 13.6±1.2, while the NTT-MMP-2 transgenics had a ratio of 20.8±2.4 (P<0.05, n = 4 for each study group).

Starting at five months of age, serial echocardiograms were performed to quantify left ventricular ejection fraction (LVEF), left ventricular end diastolic volume (LVEDV), left ventricular end-systolic volume (LVESV) and left ventricular mass in wild type and NTT-MMP-2 transgenic mice. As summarized in Figure 8, panel II., the only difference between the two study groups at 5 months of age was a small, but significant reduction in LVEF in the transgenic mice (upper left graph). By 8 months of age there was also a significant increase in the LVESV in the transgenic mice (lower left graft). By twelve months of age all assessed functional parameters were abnormal and significantly different from the wild type littermate controls. Between 12 and 15 months there was a much more rapid decline in functional parameters in the transgenic mice, with large increases in LVESV, LVEDV and LV mass, associated with clinical findings of congestive heart failure (Figure S2 depicts representative 14 month old wild type and NTT-MMP-2 transgenic mice).

### Cardiac NTT-MMP-2 expression enhances ex vivo ischemia/reperfusion injury with delayed recovery of ventricular function and more extensive myocardial infarction

**Figure 9:**
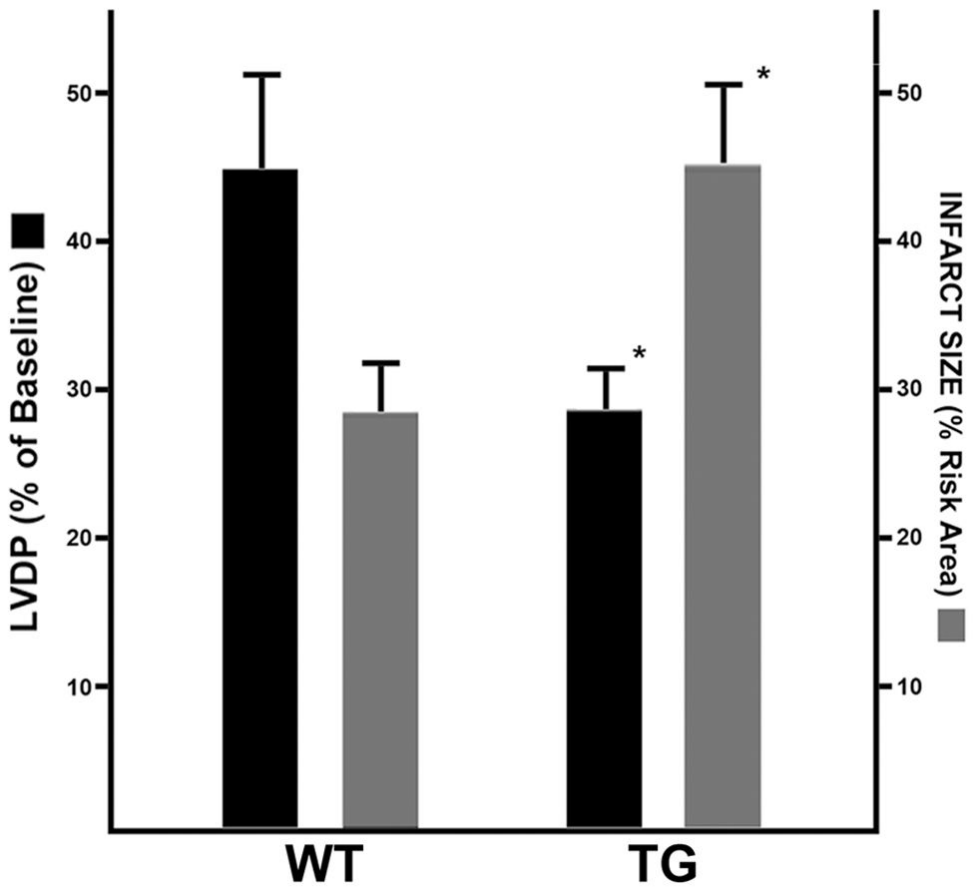
NTT-MMP-2 transgenic hearts exhibit enhanced injury following ex vivo ischemic-reperfusion injury. Isolated hearts were subjected to ex vivo ischemia/reperfusion injury (30 min/30 min) as detailed in Materials and Methods. Left ventricular developed pressure (LVDP) and infarct size were measured as detailed in Materials and Methds. Recovered LVDP, expressed as the per cent of baseline LVDP, was significantly reduced in the NTT-MMP-2 transgenic hearts. Infarction size was increased by nearly 50% in the transgenic hearts. (n = 5–6 for each study group; * P<0.05).

Ex vivo hemodynamics and induced ischemia/reperfusion injury were evaluated in four month old wild type and NTT-MMMP-2 transgenic hearts as detailed in Materials and Methods. Following 30 minutes of ischemia/30 minutes of reperfusion, the LV developed pressure (LVDP), expressed as a per cent of baseline LVDP (75±22 mm Hg), was 44±8% in the wild type hearts and 27±3% in the transgenic hearts (P<0.05, Figure 9). In addition to depressed recovery of LVDP, the transgenic hearts had a significant increase in infarct size relative to the area at risk (WT: 25.7±12.7% vs. TG: 39.6±10.0%; P<0.05, n = 5–6 for each study group, Figure 9). Thus, at an age when basal cardiac physiology was normal, the NTT-MMP-2 transgenic mice have evidence for a latent functional defect resulting in enhanced myocardial injury following experimental ischemia/reperfusion.

## Discussion

### MMP-2 is involved at multiple levels in the evolution of cardiac disease

Investigations into the role of MMP-2 in cardiac disease can be divided into three relatively discrete levels of inquiry. At the first level, researchers have focused on the pathophysiologic roles of secreted, full length MMP-2, particularly as this form relates to the regulation of the cardiac extracellular matrix compartment [17]. For example, these studies demonstrated that MMP-2 promotes ventricular rupture in the setting of experimental myocardial infarction [18]. Clinical studies of congestive heart failure have correlated outcomes (survival) and extent of ventricular remodeling with circulating levels of MMP-2 [19], [20]. More recent studies into the extracellular actions of MMP-2 have shown that cardiac-specific transgenic expression of the full length MMP-2 protein induces myxomatous mitral valve disease and coronary artery ectasia [21], [22].

At the second level, Schulz and colleagues demonstrated in a series of studies a discrete role for intracellular MMP-2 in the evolution of cardiac dysfunction, particularly during acute ischemia/reperfusion injury [23], [24]. In this setting, intracellular MMP-2 is present in a latent, full length form in close physical association with the sarcomeric contractile apparatus. Acute ischemia-reperfusion injury, with concomitant generation of reactive oxygen species and peroxynitrite, generates active MMP-2 through oxidative opening of the cysteine-switch within the MMP-2 inhibitory prodomain. This event results in MMP-2-mediated cleavage of several critical components of the contractile apparatus resulting in acute contractile dysfunction (reviewed in ref. [25]). Ali et al., [26], have recently provided a compelling cell biologic mechanism whereby an apparently conventional secreted protein, in this case full length MMP-2, can also be found in an intracellular localization. These studies demonstrated that the MMP-2 secretory sequence is relatively inefficient with respect to translocation into the endoplasmic reticulum. A substantial fraction of recently synthesized MMP-2 protein is not translocated and remains within the cell where it remains inactive until exposed to acute redox stress.

The third level of inquiry was stimulated by a detailed analysis of cardiac-specific transgenic mice expressing enzymatically active, full-length MMP-2 [4]. At the time these mice were generated, we expected that they would show alterations in the cardiac extracellular matrix due to activation of cardiac fibroblast collagen synthesis. While we did report that older transgenic mice displayed significant cardiac fibrosis, the phenotype of younger transgenic mice was characterized by prominent cardiomyocyte hypertrophy, myofilament lysis, and mitochondrial structural and functional abnormalities. As these mice aged, there was a massive induction of endogenous MMP-2 synthesis mediated by redox stress/AP-1 mediated transactivation of MMP-2 gene transcription, associated with severe systolic dysfunction [4].

The unexpected structural and functional abnormalities of the full length MMP-2 transgenic mice led to the subsequent identification and characterization of an intracellular N-terminal truncated isoform of MMP-2 generated by hypoxia and oxidative stress [1]. The NTT-MMP-2 isoform was first detected in mitochondrial-enriched ventricular extracts from older MMP-2 transgenic mice at a time in which endogenous, full length MMP-2 was induced by oxidative stress in the setting of advanced systolic failure. Subsequent *in vivo* and *in vitro* studies using model H9C2 cardiomyoblast cells demonstrated that the NTT-MMP-2 isoform was generated by oxidative stress-mediated activation of an alternate promoter within the first intron of the MMP-2 gene [1]. Microarray analyses of H9C2 cells transfected with the NTT-MMP-2 cDNA revealed three highly defined ontologies, including activation of innate immunity, apoptosis and chemokines. Further, we observed mitochondrial-nuclear stress signaling through activation of NF-κB, NFAT and IRF transcriptional cascades.

### Relationships between *in vitro* observations with NTT-MMP-2 and the NTT-MMP-2 cardiac-specific transgenic mouse

Based on our prior in vitro studies with model cardiomyoblast H9C2 cells, we hypothesized that cardiac-specific transgenic expression of the NTT-MMP-2 isoform would result in progressive cardiomyocyte and ventricular hypertrophy, cardiomyocyte apoptosis and inflammatory cell infiltration with mononuclear cells. As detailed in the Results section of this paper, the phenotype of the NTT-MMP-2 cardiac-specific transgenic mice closely matched the predicted phenotype from the in vitro studies and microarray analyses. The development of ventricular and cardiomyocyte hypertrophy observed in these mice is most probably the consequence of concurrent activation of NFAT and NF-κB transcriptional cascades. Molkentin and colleagues have reported in a series of studies on the role of NFAT transcriptional activation and the subsequent development of cardiac hypertrophy and pathological remodeling [27]–[29]. In addition, this group more recently reported on the interplay between NFAT and NF-κB activation for a coordinated development of pathologic cardiac hypertrophy [30].

While transient activation of NF-κB appears to be beneficial in cardioprotection following acute ischemia/reperfusion injury, more prolonged activation of this transcriptional network is associated with the activation of cardiac innate immunity, with initiation of chemokine and apoptotic cascades [31], [32]. Within this context, there is accumulating experimental evidence for a biphasic role of innate immunity in cardiac injury, whereby short periods of innate immunity activation are linked to cardioprotection [33]. In contrast, prolonged periods of innate immunity activation have been proposed to contribute to increased cardiomyocyte apoptosis and generation of pro-inflammatory cytokines and chemokines [33]–[35]. These are precisely the phenotypic features described in this report, which demonstrates that prolonged activation of the cardiac innate immune system, in this case through the action of NTT-MMP-2, is detrimental to cardiac structure and function. Further, as demonstrated by the enhanced degree of myocardial infarction following ex vivo ischemia-reperfusion injury, such hearts display an exaggerated innate immune response.

### The phenotype of the NTT-MMP-2 cardiac transgenic mouse is a subset of the full length MMP-2 cardiac transgenic phenotype

Prominent phenotypic features of the full length MMP-2 cardiac transgenic mice were progressive cardiomyocyte and ventricular hypertrophy associated with systolic dysfunction. Further, these hearts had mitochondrial structural abnormalities, myofilament lysis, cardiomyocyte dropout, interstitial collagen deposition and infiltration by mononuclear inflammatory cells [4]. In addition, these mice manifested latent mitochondrial abnormalities with enhanced injury following ex vivo ischemia-reperfusion injury [36]. In comparison, the NTT-MMP-2 transgenic hearts feature ventricular and cardiomyocyte hypertrophy and systolic dysfunction in the absence of extensive myofilament lysis. Further, there was no significant accumulation of interstitial collagen in these hearts, but we did observe mitochondrial structural abnormalities, mononuclear cell infiltration, cardiomyocyte apoptosis and enhanced injury responses to ex vivo ischemia-reperfusion injury.

Phenotypic features that might be assigned to the actions of the full length MMP-2 transgene include the extensive interstitial fibrosis. Hori, et al. [37] recently reported that MMP-2 directly stimulates cardiac fibroblast collagen-I expression through phosphorylation of focal adhesion kinase. Moreover, the myofilament lysis observed in the full length MMP-2 transgenic mice, but not the NTT-MMP-2 transgenic, may be the consequence of inefficient rough endoplasmic reticulum translocation of the full length MMP-2 protein, which is known to localize to the sarcomeric apparatus as detailed above. In the setting of the progressive ventricular failure and concomitant oxidative stress seen in these mice, one would anticipate opening of the cysteine switch, MMP-2 activation and sarcomere degradation.

Finally, given the localization of the NTT-MMP-2 protein to the cardiomyocyte mitochondria, it is likely that the mitochondrial structural and functional abnormalities seen in both the full length MMP-2 and NTT-MMP-2 mice are the consequence of oxidative stress-mediated activation of the MMP-2 alternate intronic promoter and generation of the NTT-MMP-2 isoform.

### Proposed model of MMP-2 in cardiac disease integrating the three levels of investigation

Based on the above considerations, we would propose the following sequence of pathophysiologic events relating MMP-2 to progressive cardiac injury: 1) As a first step, latent, full length MMP-2 associated with cardiomyocyte sarcomeres is activated by acute oxidative stress as the consequence of ischemic injury, resulting in depressed cardiac contractility. 2) As a second step, in the setting of ongoing oxidative stress, MMP-2 gene transcription is activated via the AP-1 binding site in the MMP-2 promoter, generating full length MMP-2 protein [4], [38], [39]. The bulk of this protein is secreted and participates in dysfunctional cardiac extracellular matrix remodeling, including stimulation of collagen-1 synthesis. However, a substantial fraction of this full length MMP-2 protein is not efficiently translocated across the RER and further contributes to sarcomeric (myofilament) lysis. 3) As the third step, in the setting of persistent oxidative stress, the alternative MMP-2 intronic promoter is activated and generates the NTT-MMP-2 isoform. The NTT-MMP-2 isoform activates mitochondrial-nuclear stress signaling (NFAT, NF-κB, IRF cascades) and induces pathologic cardiomyocyte and ventricular hypertrophy (NFAT, NF-κB). Furthermore, mitochondrial structural and functional abnormalities develop, in association with myocardial inflammation and cardiomyocyte apoptosis. The final consequence of this course of events is progressive cardiac failure and death.

### Summary and potential clinical implications

We conclude that a discrete isoform of MMP-2 generated by oxidative stress-mediated activation of an alternate intronic promoter in the MMP-2 gene, in conjunction with the actions of the full length MMP-2 isoform, collectively contribute to the complex cellular and functional phenotypes characteristic of progressive ischemic cardiomyopathy. Our findings suggest that the relative contributions of these two MMP-2 isoforms are temporally distinct and that the NTT-MMP-2 isoform is a downstream mediator of a discrete subset of the phenotypic features characteristic of ischemic cardiomyopathy. In spite of some clinical trial reversals, recent studies of non-selective MMP inhibitors and anti-oxidants suggest that MMP-2 remains a compelling pharmacologic target for the treatment of cardiovascular disease [41], [42].

## Materials and Methods

The investigation was approved by the Animal Care and Use Subcommittee (IACUC) of the San Francisco Veterans Affairs Medical Center (protocol 09-053-03) and conformed with the *Guide for the Care and Use of Laboratory Animals* published by the National Institutes of Health (NIH Publication 85–23, Revised 1996). This institution is accredited by the American Association for the Accreditation of Laboratory Animal Care (Institutional PHS Assurance Number is A3476-01).

### Construction of N-terminal truncated (NTT) MMP-2 cDNA

The cDNA encoding the full-length human MMP-2 protein was obtained from Origene. Using the full length MMP2 cDNA as a template, NTT-MMP2 was generated with sense primer, 5′-TGCAAGCTTTTGT-GCTGAAAGATACC3′, and antisense primer, 5′-CCTCTAGACTCGAGCGGC-3′. This generated a NTT-MMP-2 cDNA construct (cloned into pcDNA3.1, Invitrogen) starting at base pair +81 relative to the ATG encoding M^1^ of the full length MMP-2 protein. The native Kozak consensus sequence flanking amino acid M^77^ (aagAagA_+229_TGc) was not modified. The NTT-MMP-2 cDNA was subcloned into the EcoR1 site of pEGFP-N1 (Clontech), to create an expression cassette consisting of the N-terminal truncated MMP-2 in frame with C-terminal EGFP.

A NTT-MMP-2/eGFP protein positive control was generated by transient transfection of the NTT-MMP-2/EGFP expression plasmid into CHO cells (ATCC) using standard methodology. At 48 hours following transfection, CHO cells were lysed into 50 mM Tris/HCl, pH 7.4, 150 mM NaCl, 0.5% Triton X-100, 0.5% CHAPS, 0.5% sodium deoxycholate, plus protease inhibitor cocktail (Pierce). The NTT-MMP-2/EGFP protein was recovered by affinity chromatography on gelatin-coupled Sepharose (Sigma) as reported [40].

### Generation of cardiac-specific N-terminal truncated MMP-2 transgenics

The NTT-MMP/EGFP expression cassette was isolated by PCR to add Sal I and Hind III restriction sites, followed by cloning into these restriction sites in the α-myosin heavy chain promoter vector (the kind gift of Dr. Jeffrey Robbins). The assembled construct was excised by Not I digestion, purified and microinjected into 129Sv x CD1 background fertilized pronuclei. Transgenic mice were identified by PCR of tail genomic DNA using a 5′ primer for MMP-2 (5′-TGGATCCTGGCTTCCCC-AAGCTCATCG-3′) and a 3′ primer for EGFP (5′-GCTGAAGCACTGCACGCCGTAGGTCA-3′). Nine founders were identified and three independent lines were evaluated as detailed below. The transgenic lines were maintained as heterozygotes in the outbred CD1 background using wild type CD1 mice obtained from Charles River. All experiments were performed by comparing the transgenic mice with their wild type litter mate controls.

### MMP-2 transgene expression

Hearts of euthanized mice were perfused in situ with 4°C PBS until free of blood. Excised hearts were homogenized in 0.25 M sucrose, 10 mM HEPES, pH, 7.5, 5 mM EDTA and protease inhibitor cocktail at 4° C, followed by centrifugation at 700 g for 5 minutes. The supernate was centrifuged for an additional 5 minutes at 700 g and the resulting supernate centrifuged at 9000 g for 5 minutes to pellet mitochondria. The mitochondrial pellets were homogenized in lysis buffer (50 mM Tris/HCl, pH 7.4, 150 mM NaCl, 0.5% Triton X-100, 0.5% CHAPS, 0.5% sodium deoxycholate, plus protease inhibitor cocktail), sonicated briefly on ice and the supernate collected after centrifugation at 10,000 g for 20 minutes. Extracts (150 μg protein/sample) were incubated overnight at 4°C with 100 μl gelatin-Sepharose beads (Sigma-Aldrich) in 500 μl 50 mM Tris/HCl, pH 7.4 to affinity absorb MMP-2. Thereafter, the beads were washed three times in binding buffer, followed by elution in an equal volume of 2 X SDS-PAGE sample buffer. Western blots used murine monoclonal anti-MMP-2 (Ab-3, Calbiochem) followed by HRP-conjugated goat anti-mouse IgG (Zymed) and detection with ECL-Plus reagent.

### Histologic methods

Mice were anesthetized with ketamine/xylazine and arrested in diastole by intraventricular injection of 4°C 50 mM KCl. The hearts were then perfused for 30 minutes at 20 mm Hg pressure with ice-cold buffered 4% paraformaldehyde, followed by paraffin embedding. Five micron sections were stained with hematoxylin/eosin or Picrosirius red using standard methodology. Cardiomyocyte cross-sectional areas were determined as reported [4]. Intracellular localization of the MMP-2-EGFP transgene was performed on deparaffinized sections following citrate antigen retrieval (Vector) using a 1∶1000 dilution of rabbit polyclonal anti-GFP (Abcam, Ab6556) for four hours, followed by incubation with 1∶2000 biotinylated goat-anti-rabbit IgG. Histochemical development with diaminobenzidine-Ni^+2^ (DAB- Ni^+2^, Vector) was restricted to three minutes to limit reaction product migration, followed by a hematoxylin counterstain. Images were digitally captured at high resolution (4000 dpi). A dense focus of DAB- Ni^+2^ reaction product in the transgenic image was sampled in Adobe Photoshop CS4. Foci with similar pixel densities were converted to a fluorescent green pseudocolor using the Image Adjust/Change Color tool to enhance contrast with background DAB- Ni^+2^ staining. The wild type control images were processed simultaneously in an identical fashion. The original digital image is shown as Figure S1.

Assessment of cardiomyocyte myofibrillar degeneration was performed with Luxol Fast Blue staining as detailed [15], [16]. Cardiac cross-sectional areas were determined as previously reported [4].

For transmission electron microscopy, hearts were perfused with ice-cold phosphate-buffered saline as detailed above and blocks of the left ventricular free wall were fixed in modified Karnovsky's solution. Ultrathin sections were stained with lead citrate/uranyl acetate using standard methodology.

### Echocardiography

Transthoracic echocardiography was performed with a commercially available system (Acuson Sequoia c256, Acuson, Siemens) using a 15-MHz linear array transducer as reported. The LV end-diastolic dimension (LVEDD) and end-systolic dimension (LVESD) were determined as the largest and smallest dimensions of the LV, respectively, on M-mode images, and fractional shortening (FS) was derived from following equation: FS  =  (LVEDD minus LVESD)/LVEDD. The LV end-diastolic volume (LVEDV) was calculated using the following two-dimensional area-length method: LVEDV  = (5/6) A minus L, where A is the endocardial parasternal short-axis area at end-diastole and L is the parasternal long-axis length.

Comparisons were made at each time point using an unpaired t-test. P<0.05 was considered significant.

### Ischemia/reperfusion protocol and *ex vivo* hemodynamics

Mice were anesthetized with sodium pentobarbital (60 mg/kg, IP) and anticoagulated with heparin sodium (5000 USP U/kg, IP).

Hearts were rapidly excised and washed in ice-cold NaCl (120 mm/l) and KCl (30 mm/l) and cannulated via the aorta on a 20 gauge stainless steel blunt needle. Hearts were then perfused at a constant pressure of 70 mm Hg on a Langendorff rig using Krebs-Henseleit solution containing 5 mmol/l glucose and 5 mmol/l sodium pyruvate bubbled with 95% O_2_-5% CO_2_ at 37°C. A stimulus generator connected to platinum electrodes was used to pace the hearts at 360 bpm. Ischemia/reperfusion injury of isolated murine hearts and assessment of hemodynamics was performed as previously reported [36]. After baseline hemodynamics were recorded during a 20 min equilibration period, hearts were subjected to 30 min of global ischemia and 30 min of reperfusion. Upon completion of reperfusion, hearts were perfused with 1% 2,3,5-triphenyltetrazolium chloride solution and fixed in 10% neutral buffered formalin. The left ventricle was sliced into transverse sections and each section was weighed. Both sides of each section were imaged with a color digital videocamera (Leica, COHU Y/C 460 HTYL, 768×494 array, San Diego CA) connected to a microscope (Leica, Stereo Zoom 6 photo, San Diego CA). Images of the viable areas (red-stained) and necrotic areas (unstained) were analyzed using NIH Scion Image software in a blinded fashion. Infarct size was adjusted to the weight of each section and expressed as a percentage of total left ventricular mass. Comparisons between WT and MMP-2 transgenic mice were made using Students *t*-test and two-way repeated measures ANOVA where values of *P*<0.05 were considered statistically significant.

## Supporting Information

Figure S1 **Original** (**non-pseudocolor**) **image of NTT-MMP-2/eGFP transgenic hearts stained for eGFP protein.** (x325).
(TIF)

Figure S2 **Wild type and NTT-MMP-2/eGFP transgenic mice at fourteen months of age.** The transgenic mouse has evident signs of congestive heart failure, including prominent ascites and edema.
(TIF)
