# Supplementary figures and images for: N-Terminal Truncated Intracellular Matrix Metalloproteinase-2 Induces Cardiomyocyte Hypertrophy, Inflammation and Systolic Heart Failure
Source: PLoS One. 2013 Jul 16;8(7):e68154. doi: 10.1371/journal.pone.0068154 (PMC3712965; doi:10.1371/journal.pone.0068154)

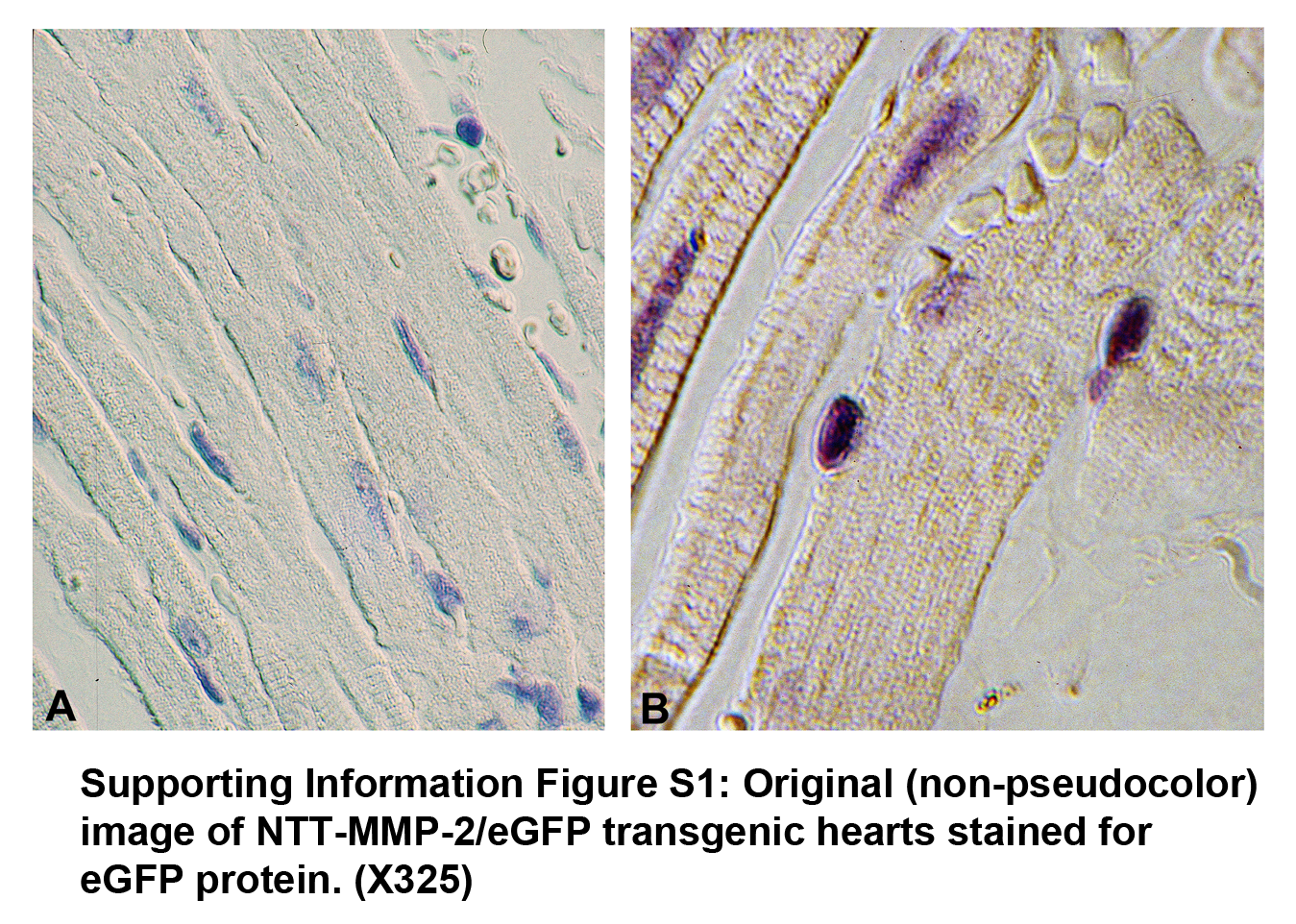

Supplement: Figure S1 — Original (non-pseudocolor) image of NTT-MMP-2/eGFP transgenic hearts stained for eGFP protein. (x325). (TIF) [file pone.0068154.s001.tif]

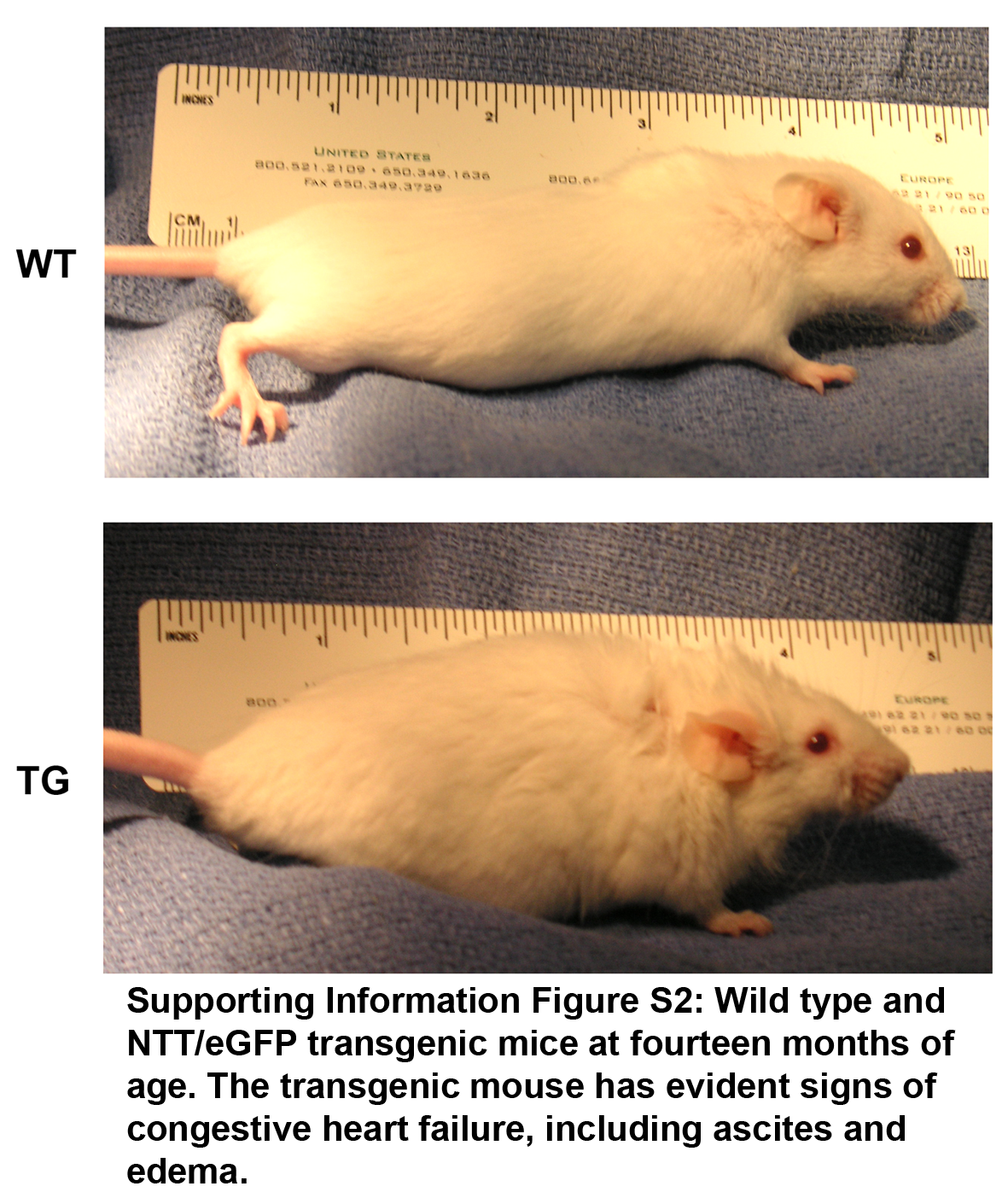

Supplement: Figure S2 — Wild type and NTT-MMP-2/eGFP transgenic mice at fourteen months of age. The transgenic mouse has evident signs of congestive heart failure, including prominent ascites and edema. (TIF) [file pone.0068154.s002.tif]
